# Takotsubo Cardiomyopathy as an Early Complication of Drug-Induced Suicide Attempt

**DOI:** 10.1155/2013/946378

**Published:** 2013-11-14

**Authors:** Massimo Romanò, Federica Zorzoli, Roberta Bertona, Rosvaldo Villani

**Affiliations:** Department of Cardiology, Vigevano Hospital, Corso Milano 19, 27029 Vigevano, Italy

## Abstract

Takotsubo cardiomyopathy typically presents in menopausal women following episodes of intense physical or mental stress. To our knowledge, the literature contains only two documented cases of Takotsubo cardiomyopathy arising following a suicide attempt, neither of which involved pharmaceutical poisoning. Here, however, we document the case of a young male patient with borderline personality disorder and a clinical and angiographic presentation compatible with Takotsubo cardiomyopathy arising following a suicide attempt by voluntary drug intoxication (risperidone, barbiturates, and benzodiazepine). The potential pathophysiological mechanisms behind this unusual clinical picture are discussed.

## 1. Introduction

Takotsubo syndrome, or stress-induced cardiomyopathy, manifests as a transitory dysfunction of the left ventricle, whose characteristic angiographic appearance is known as apical ballooning. It mimics the symptoms and electrocardiographic appearance of acute myocardial infarction, generally with ST segment elevation but without any significant signs of coronary lesions under angiography. Its pathogenesis is probably secondary to a damage to the myocytes mediated by the catecholamines and microvascular dysfunction [1].

Over recent years, increasing numbers of cases, arising in various circumstances of physical or mental stress, have been described in the literature. Nonetheless, only two reported cases brought on following a suicide attempt have been documented, one following the consumption of herbicide [2] and the other by hanging [3]. To our knowledge, this is the first reported case of Takotsubo cardiomyopathy arising following a suicide attempt by ingestion of psychotropic drugs. 

## 2. Case Report

A 40-year-old male with type 2 diabetes mellitus and obesity (110 kg, 172 cm, and BMI 37.2) who underwent a biliodigestive bypass in 2011 was brought in a comatose state to our accident and emergency department by the emergency services. This followed a suicide attempt, attested to by a note addressed to family members, by ingestion of phenobarbital, risperidone, lorazepam, clotiazepam, and bisoprolol. The patient was already known to the mental health services as a sufferer of borderline personality disorder and was being treated with 5 mg/day olanzapine.

**Figure 1:**
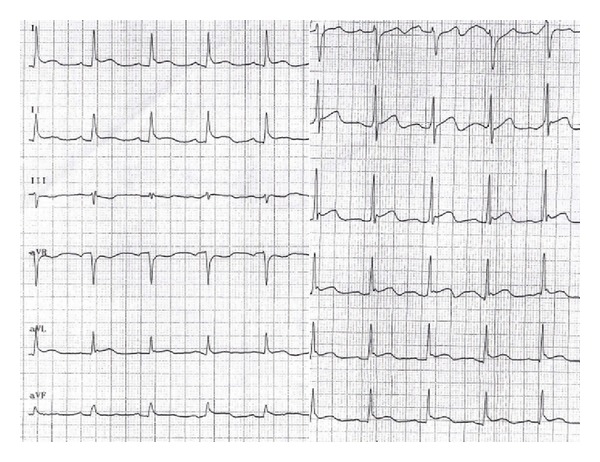
ECG on admission, showing anterolateral ST segment elevation.

**Figure 2:**
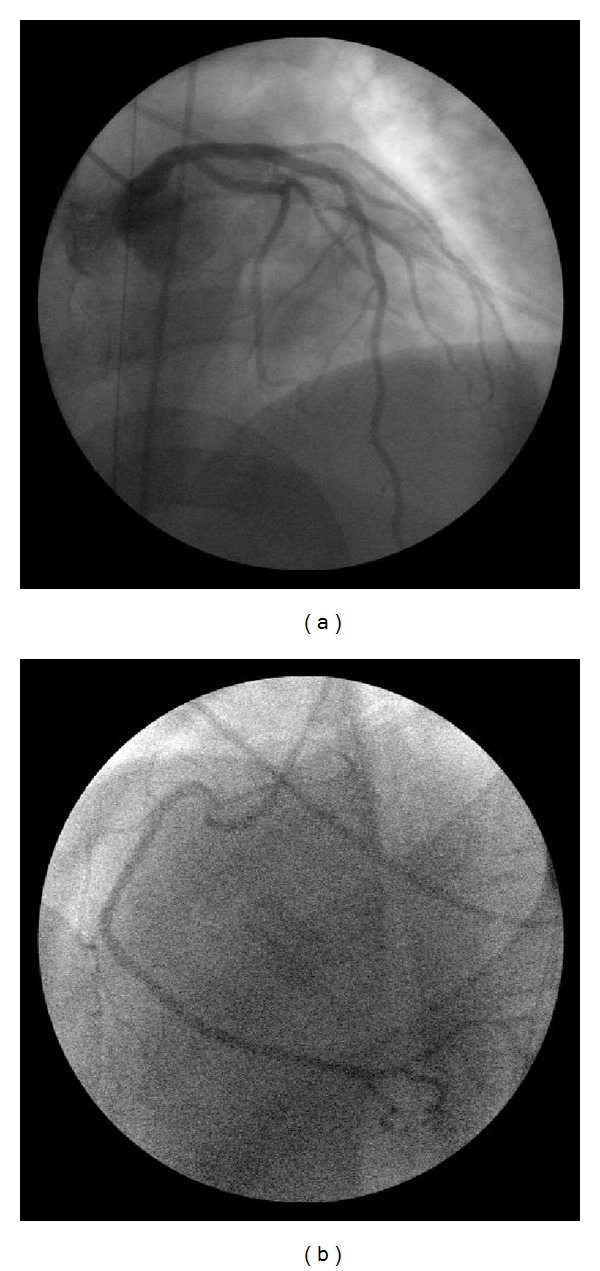
Left (a) and right (b) coronary arteries, without significant coronary lesions.

**Figure 3:**
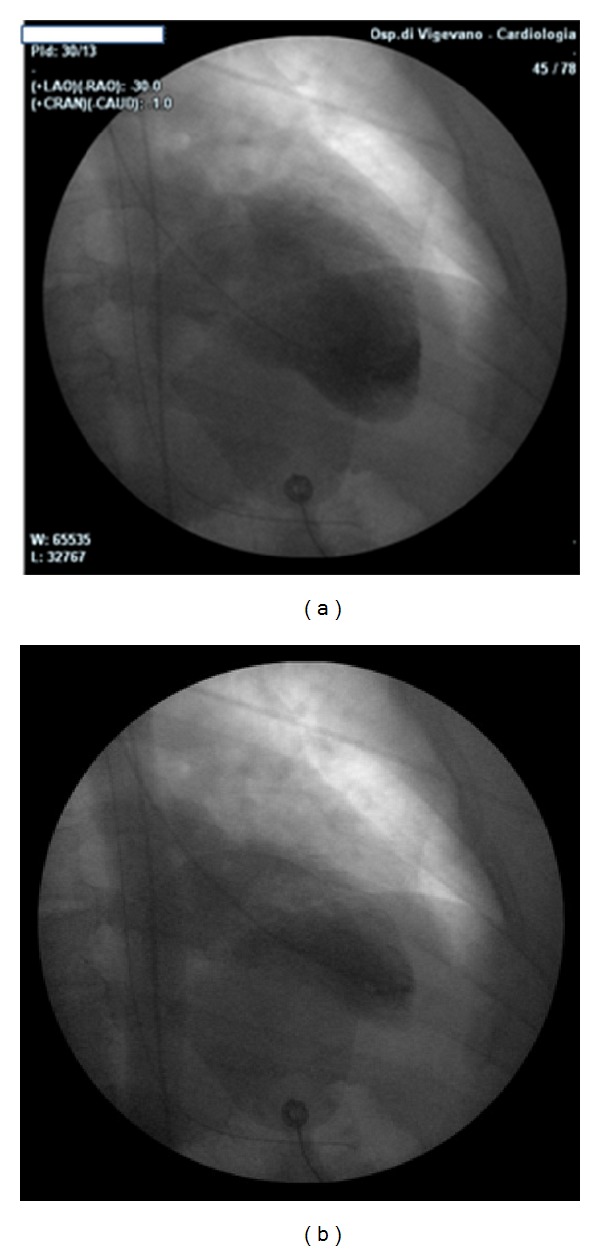
Left ventriculography in diastole (a) and systole (b), showing the apical hypokinesis.

Tests conducted on the patient in accident and emergency confirmed the comatose state, Glasgow Coma Scale 5, and revealed an arterial pressure of 110/80 mmHg, heart rate 80 bpm, and oxygen saturation at 94%, with an oxygen mask set at 6 L/min. Arterial blood gas analysis showed respiratory acidosis, and the patient was fitted with a nasogastric tube and orotracheal intubation. ECG showed anterolateral ST elevation (Figure 1), while an echocardiogram showed normal ventricular chamber dimensions, apical akinesis, and a slight overall reduction in contractile function of the left ventricle (ejection fraction 0.50). The patient therefore underwent emergency coronary angiogram, which revealed normal coronary arteries (Figure 2). Left ventriculography confirmed the marked apical hypokinesis (Figure 3).

The patient, still on mechanical ventilation, was moved to our intensive care unit, where his haemodynamic status remained stable. Toxicological examination revealed high serum concentrations of phenobarbital (50 mcg/mL, normal values 10–40 mcg/L) and risperidone (149 mcg/mL, normal values < 100 mcg/mL), and concentrations of lorazepam and clotiazepam within the normal range. Myocardial damage markers were not significantly elevated (troponin T Hs 15 pg/mL, normal values < 11 pg/mL). 

The patient was treated with ramipril by the oral route, starting on 5 mg/day and rising incrementally up to 10 mg/day. Treatment was completed with bisoprolol at 1.25 mg/day, together with aspirin 100 mg and enoxaparin 6000 U twice daily.

**Figure 4:**
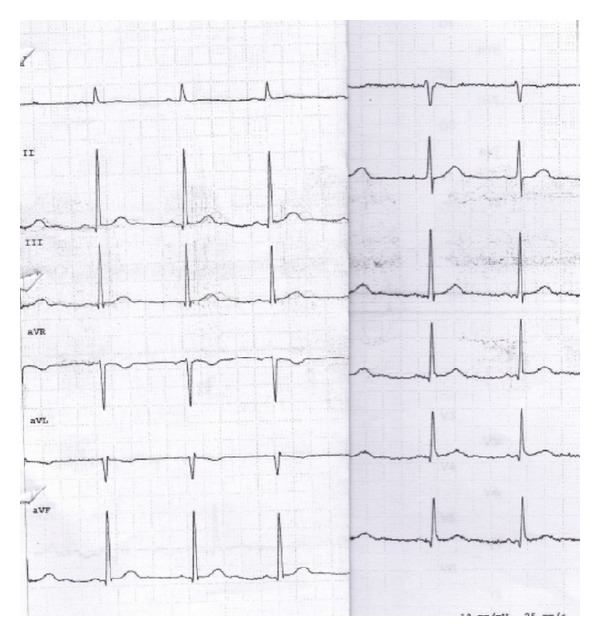
ECG at discharge, showing normal ST segment.

On the third day, the breathing tube was removed and the haemodynamic status remained stable at AP 140/80 mmHg, heart rate 74/bpm. The patient was therefore transferred to our Intensive Coronary Care Unit, where he recovered with no complications, and his electrocardiogram returned to normal (Figure 4). 

Echocardiography performed on the sixth day of hospitalization showed that the segmental kinesis had returned to normal, with ejection fraction 0.70. On the seventh day, the patient was transferred to the psychiatric unit, from which he was discharged after 10 days. 

## 3. Discussion

Takotsubo syndrome was first described by Sato et al. in 1990 [4], who named it after the morphology, peculiar to this condition, of the left ventricle, whose apex takes on the appearance of a balloon with a narrow neck, a shape reminiscent of the basket used by Japanese fishermen to catch octopi. Over the years, cases of stress-induced dysfunction of the left ventricle with alterations in the kinesis of the anterolateral segments but normal contractile apex have also been described [5]. 

According to a large survey in the USA [6], the prevalence of Takotsubo cardiomyopathy was 0.02% of all hospital admissions in the USA in 2008. The majority of these cases were seen in middle-aged women with a history of smoking, excess alcohol consumption, states of anxiety, and hyperlipidaemia. The in-hospital mortality is reported as roughly 4.2% [7]. 

The pathophysiological mechanisms behind Takotsubo syndrome are still the object of discussion, although a multifactorial origin is the most popular theory. That being said, alterations in the coronary microcirculation, vasospasm, neurogenic stunning caused by acute autonomic dysfunction, and catecholaminergic cardiotoxicity have all been implicated [8–11]. This latter hypothesis is supported by cases of left ventricular dysfunction in patients with pheochromocytoma [10], but not all studies have shown high blood levels of noradrenaline. It is therefore possible that the density and activity of beta-adrenergic receptors play a role in the left ventricular dysfunction. 

Numerous studies have also shown correlations between several psychiatric illnesses and a high incidence of cardiovascular events [11]. In particular, psychiatric disturbances associated with anxiety and posttraumatic stress seem to have a greater correlation with cardiovascular events than depressive disorders. A higher-than-normal incidence of cardiovascular events has also been correlated with personality disorders [12], like the borderline patient described here, a 40-year-old male receiving neuroleptic treatment. It is likely that common underlying pathophysiological mechanisms such as sympathetic nervous system alterations, reduced heart rate variability, altered platelet function and an increase in pro-inflammatory processes play a role in Takotsubo onset. Furthermore, the catecholaminergic system is also the most affected following exposure to trauma [13].

It has been observed, in a large retrospective study of Takotsubo syndrome patients, a higher prevalence of depression, compared with a group of patients with acute anterior myocardial infarction [14]. Nevertheless, 4 cases of Takotsubo syndrome associated with an exacerbation of psychiatric illness have been described in the literature, all in women aged between 53 and 67 years. Respectively, these women had a diagnosis of Alzheimer's with psychotic features, adjustment disorder, major depressive disorder, and type 1 bipolar disorder [15]. 

There are, however, only two previous cases of Takotsubo syndrome arising following a suicide attempt reported in the literature, one by glufosinate ammonium herbicide intoxication [2] and the second by hanging [3]. We hypothesize that in our patient, the only known documented case arising following attempted suicide by psychiatric drug ingestion, Takotsubo cardiomyopathy may have been triggered by an exacerbation of his psychiatric illness, which features high baseline levels of catecholamines that could have been further elevated by the stress experienced during the suicide attempt.
